# A microfluidic diagnostic device with air plug-in valves for the simultaneous genetic detection of various food allergens

**DOI:** 10.1038/s41598-022-16945-2

**Published:** 2022-07-27

**Authors:** Daigo Natsuhara, Sae Misawa, Ryogo Saito, Koki Shirai, Shunya Okamoto, Moeto Nagai, Masashi Kitamura, Takayuki Shibata

**Affiliations:** 1 Department of Mechanical Engineering, Toyohashi University of Technology, Toyohashi, Aichi 441-8580 Japan; 2 Faculty of Pharmacy and Pharmaceutical Sciences, Josai University, Sakado, Saitama 350-0295 Japan

**Keywords:** Biological techniques, Health care, Engineering

## Abstract

The identification of accidental allergen contamination in processed foods is crucial for risk management strategies in the food processing industry to effectively prevent food allergy incidents. Here, we propose a newly designed passive stop valve with high pressure resistance performance termed an “air plug-in valve” to further improve microfluidic devices for the detection of target nucleic acids. By implementing the air plug-in valve as a permanent stop valve, a maximal allowable flow rate of 70 µL/min could be achieved for sequential liquid dispensing into an array of 10 microchambers, which is 14 times higher than that achieved with the previous valve arrangement using single-faced stop valves. Additionally, we demonstrate the simultaneous detection of multiple food allergens (wheat, buckwheat, and peanut) based on the colorimetric loop-mediated isothermal amplification assay using our diagnostic device with 10 microchambers compactly arranged in a 20-mm-diameter circle. After running the assays at 60 °C for 60 min, any combination of the three types of food allergens and tea plant, which were used as positive and negative control samples, respectively, yielded correct test results, without any cross-contamination among the microchambers. Thus, our diagnostic device will provide a rapid and easy sample-to-answer platform for ensuring food safety and security.

## Introduction

Food allergies are immune-mediated adverse responses triggered by the inhalation or ingestion of proteins in specific foods^1–3^. The incidence and prevalence rates of food allergies have increased in recent decades, posing a global health problem. Allergens can cause not only acute allergic reactions, such as skin (hives), respiratory tract (wheezing, coughing), and gastrointestinal tract (nausea, vomiting, and diarrhea), but also critical conditions, such as anaphylaxis^4,5^. At present, food allergen labeling is mandatory and legislated in many countries. In Japan, food allergen labeling regulations have been adopted for processed foods containing seven specific raw materials, including wheat, buckwheat, peanut, egg, milk, shrimp, and crab. The guidelines stipulate that any foods containing allergenic proteins must be labeled if the concentration exceeds 10 mg/kg (ppm)^6^. For food-allergic consumers, even a tiny quantity of an allergen can induce an allergic reaction^7^. Therefore, the rapid and easy identification of processed foods accidentally contaminated with allergenic substances is crucial for risk management strategies in the food processing industry to effectively prevent food allergy incidents.

Many methods for detecting various food allergens have been assessed^8^. The enzyme-linked immunosorbent assay (ELISA) is widely used for the quantitative analysis of targeted proteins owing to its advantages of high sensitivity, high specificity, ease of use, and high sample throughput. Accordingly, it is one of the most commonly used assays for detecting and quantifying food allergens in the food industry and in official food control agencies^9,10^. Several ELISA-based immunodiagnostic test kits are commercially available for the detection of food allergens^11–13^. However, proteins are susceptible to unintended proteolytic degradation during the extraction processes used in protein-based detection methods, which can cause false-negative results, and complex food matrices can contain interfering organic components, which may give rise to false positives^14,15^. Because DNA molecules are more stable than proteins, nucleic acid-based detection methods, among which the real-time quantitative polymerase chain reaction (qPCR) is the most widely used method for amplifying specific nucleic acid (DNA/RNA) targets, are increasingly used in food allergen detection^16–19^. However, qPCR testing requires highly trained personnel and expensive equipment (i.e., a thermal cycler) with precise temperature setpoint control for amplification and therefore is not a cost-effective and easy-to-use detection tool.

Loop-mediated isothermal amplification (LAMP) has been recognized as an alternative to qPCR for on-site detection without requiring specialized equipment^20,21^. The LAMP assay, in which a set of four to six specifically designed LAMP primers and *Bst* DNA polymerase with high strand-displacement activity are used to amplify a specific DNA target, can be performed in 30–60 min under isothermal conditions (60–65 °C)^22,23^. Various methods for detecting LAMP amplification products are available, such as visualization of the amplification products through traditional gel electrophoresis post amplification, real-time measurement of the turbidity caused by the magnesium pyrophosphate that is produced as a reaction byproduct, and real-time detection of the LAMP amplicons using an intercalating fluorescent dye (e.g., SYBR Green I). In addition, naked-eye colorimetric detection by using indicator dyes is possible. For example, phenol red is a pH-sensitive indicator dye used to monitor significant pH changes from alkaline to acidic associated with the LAMP reaction and hydroxynaphthol blue (HNB) is a metal indicator dye used to monitor a rapid decrease in the concentration of free Mg^2+^ ions owing to the formation of insoluble magnesium pyrophosphate during DNA amplification^24–26^. However, there are fundamental issues with the conventional LAMP method that remain to be solved for its application in the on-site multiplexed diagnostics of multiple food allergens. That is, it is necessary to prepare and individually test many sample/reagent mixtures for each food allergen target. This procedure requires not only long and tedious sample preparation to yield a test result, but also a relatively large amount of LAMP reagents, generally, 10–25 µL for testing a single food allergen. To address these concerns, Lab-on-a-chip technologies have considerable potential for enabling multiplexed LAMP assays in microfluidic devices for the simultaneous detection of targeted nucleic acids in a single operation^27–34^.

In our previous studies^35–37^, we developed a versatile microfluidic device for the multiplexed detection of targeted nucleic acids based on the LAMP method. The microfluidic device allows sequential dispensing of sample/reagent mixtures into an array of reaction microchambers with a single operation, thus possibly providing a platform for rapid and easy sample-to-answer diagnostics. With the aim to minimize crop losses and to ensure a stable supply of affordable food in agriculture, we demonstrated the ability of the fabricated diagnostic device to detect not only a DNA plant virus infecting tomato, but simultaneously also four RNA plant viruses infecting cucurbits within 60 min^35^. In addition, we demonstrated that the toxic plant *Colchicum autumnale* could be detected within 60 min to provide a reliable screening method for plant poisoning in emergency medical care^36^. Moreover, we can simultaneously diagnose coronavirus disease and other infectious diseases, including those caused by severe acute respiratory syndrome, seasonal influenza A, and pandemic influenza A 2009, by running a colorimetric reverse transcription LAMP assay for 30 min using our microfluidic device^37^. However, in the sequential liquid dispensing method previously proposed, the maximal allowable flow rate for dispensing multiple microchambers is limited by an increase in the flow resistance, which increases in proportion to not only the flow rate, but also the number of microchambers filled. In this study, we designed a new valve configuration to be used as a permanent stop valve (hereinafter referred to as an “air plug-in valve”) in the microfluidic diagnostic device with the aim of significantly improving the performance of sequential liquid dispensing into an array of reaction microchambers. The most distinctive feature of the air plug-in valve proposed here is that the liquids reaching a set of two passive valves push against each other through the air plug, resulting in high pressure resistance performance because the applied pressure is offset. Using an optimally designed microfluidic device with 10 microchambers arranged in a circle, we demonstrate the simultaneous detection of three food allergens based on specific DNA targets of wheat (*Triticum aestivum*), buckwheat (*Fagopyrum esculentum*), and peanut (*Arachis hypogaea*).

## Methods

### Fabrication process of the multiplexed diagnostic device

**Figure 1:**
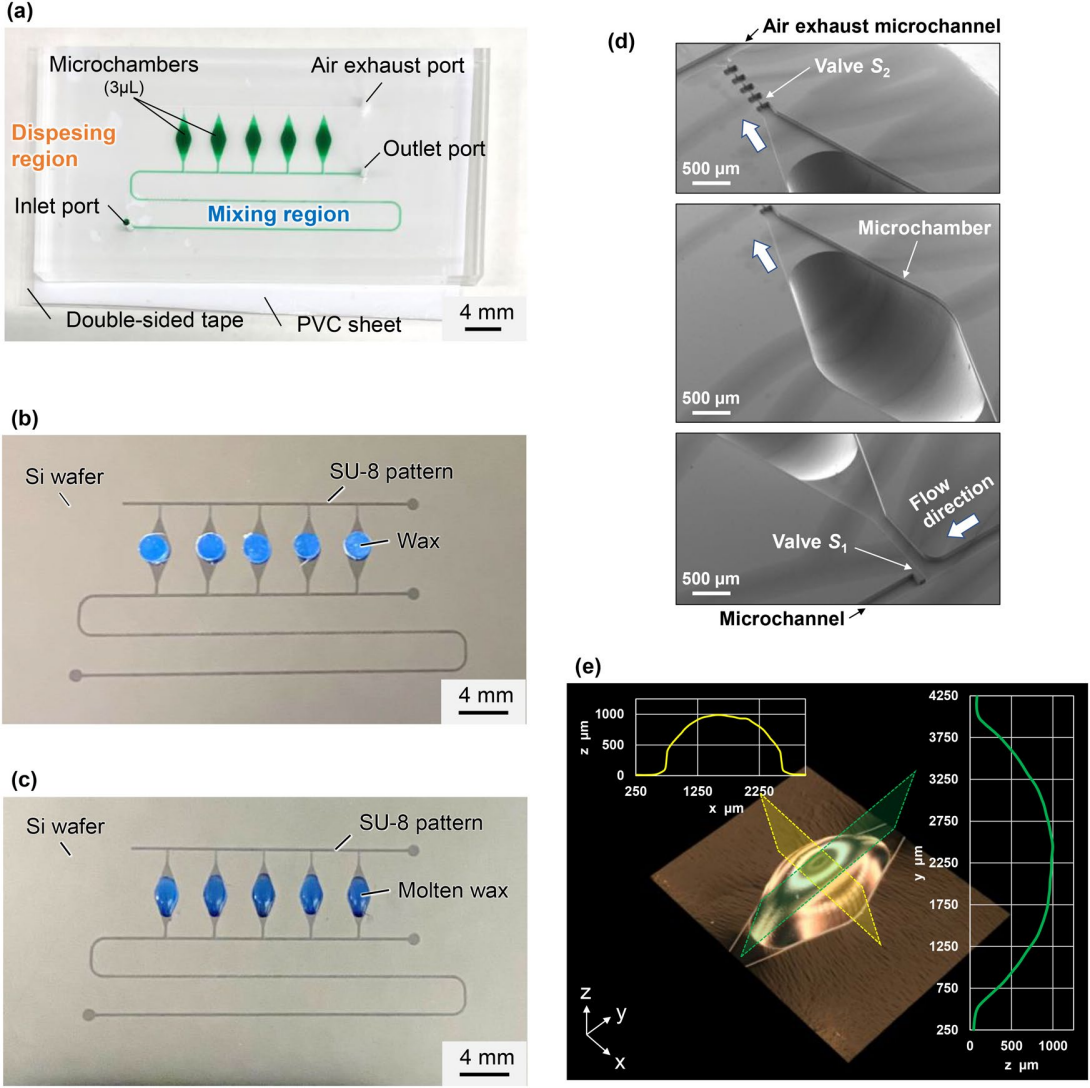
Schematic representation of the microfluidic diagnostic device allowing sequential liquid dispensing for multiplexed genetic allergen detection and fabricated using a combination of a soft lithography process and a wax reflow process. (**a**) A fabricated PDMS microfluidic device consisting of an array of five reaction microchambers (~ 3 µL each) was filled with green-colored water. (**b**) A piece of wax was placed on the top surface of each SU-8 chamber mold pattern. (**c**) Semi-ellipsoid-shaped wax molds formed with a uniform shape after heating at 135 °C for 3 min. (**d**) Scanning electron microscopy images of the PDMS microchamber. (**e**) 3D image and cross-sectional profiles of the PDMS microchamber.

We developed a fabrication process of a polydimethylsiloxane (PDMS)-based microfluidic device employed for multiplexed genetic detection, combining a soft lithography process and a wax reflow process^38^. Figure 1a shows an example of the fabricated microfluidic device (with a size of 50 mm × 25 mm) consisting of two main parts: a mixing region and a dispensing region, which also serve as the reaction and detection regions. Five microchambers in an array are interconnected via two independent rectangular microchannels (200 µm in width and 50 µm in height) for introducing the liquid into the microchambers and for exhausting the air in the microchambers, respectively.

The fabrication process used can be briefly described as follows: first, a negative thick photoresist (SU-8 3050; MicroChem, Newton, MA, USA) was patterned on a 4-inch single-crystal silicon wafer (e-Prize, Yokohama, Japan) as a mold in a single-step photolithography process. Next, to create deep localized microchamber structures (max. 1 mm in depth), the SU-8 mold and pieces of wax of 2.7 mg (Ferris File-A-Wax; Freeman Manufacturing & Supply, Avon, OH, USA) were simultaneously subjected to a low-pressure air plasma surface treatment in a plasma asher (JPA300; J-Science Lab, Kyoto, Japan) at a power of 150 W for 2 min. Then, the pieces of wax were placed in the center of the top surface of each SU-8 chamber pattern (Fig. 1b). Subsequently, a reflow process was conducted by heating the mold containing the wax pieces on a hotplate at 135 °C for 3 min (EC1200-N; AS ONE, Osaka, Japan). After cooling down to room temperature, semi-ellipse-shaped chamber molds with a uniform shape were obtained because of the surface tension of the molten wax (Fig. 1c). It should be noted that the plasma surface treatment of the SU-8 mold and the pieces of wax before the reflow process remarkably improved the adhesion strength between them, probably due to the removal of surface contaminations. Subsequently, the SU-8 master mold with the semielliptical wax structures was replicated in PDMS (Silpot 184; Dow Corning Toray, Tokyo, Japan) after curing on a hot plate at 80 °C for 40 min. After peeling off the PDMS from the SU-8 master mold, circular holes (with a diameter of 1.0 mm) for one inlet and two outlet ports were punched into the PDMS microfluidic device using a biopsy punch piercing tool (Kai Industries, Gifu, Japan). Finally, both the microchambers and microchannels were sealed with a white polyvinyl chloride (PVC) sheet (EB-235; Hikari, Osaka, Japan) using a silicone-based adhesive double-sided tape (No. 5303 W; Nitto Denko, Osaka, Japan). Figure 1d shows images of a PMDS microchamber acquired with a scanning electron microscope (S-3000 N; Hitachi High-Tech, Tokyo, Japan). A three-dimensional (3D) PDMS microchamber with an extremely smooth curved surface was obtained. A 3D image and cross-sectional profiles of the PDMS microchamber acquired with a digital microscope (VHX-7000; Keyence, Osaka, Japan) revealed that the maximum depth of the semielliptical PDMS microchamber was approximately 1 mm, the expected design value (Fig. 1e).

In this study, microfluidic devices with 10 microchambers arranged in a single row or in a circle were also fabricated by the same process. Additionally, to investigate the effect of air leakage at the interface of the PDMS device and the silicone-based adhesive double-sided tape, we prepared a modified PDMS device that was covalently bonded to a thin PDMS layer spin-coated on a 4-inch glass wafer (AS-4; Toshin Riko, Tokyo, Japan) by air plasma surface treatment. In experiments, a syringe pump (YSP-201; YMC, Kyoto, Japan) or a pressure-driven micropump (Flow EZ 7000 mbar, Fluigent SA, Le Kremlin-Bicêtre, France) equipped with a flow sensor (Flow Unit, Fluigent SA) were used to introduce the liquid in the devices. A smartphone camera was used to capture video images of the sequential liquid dispensing process into multiple microchambers and photographs of multiple microchambers to analyze color change after LAMP assays.

### Theory for sequential liquid dispensing

**Figure 2:**
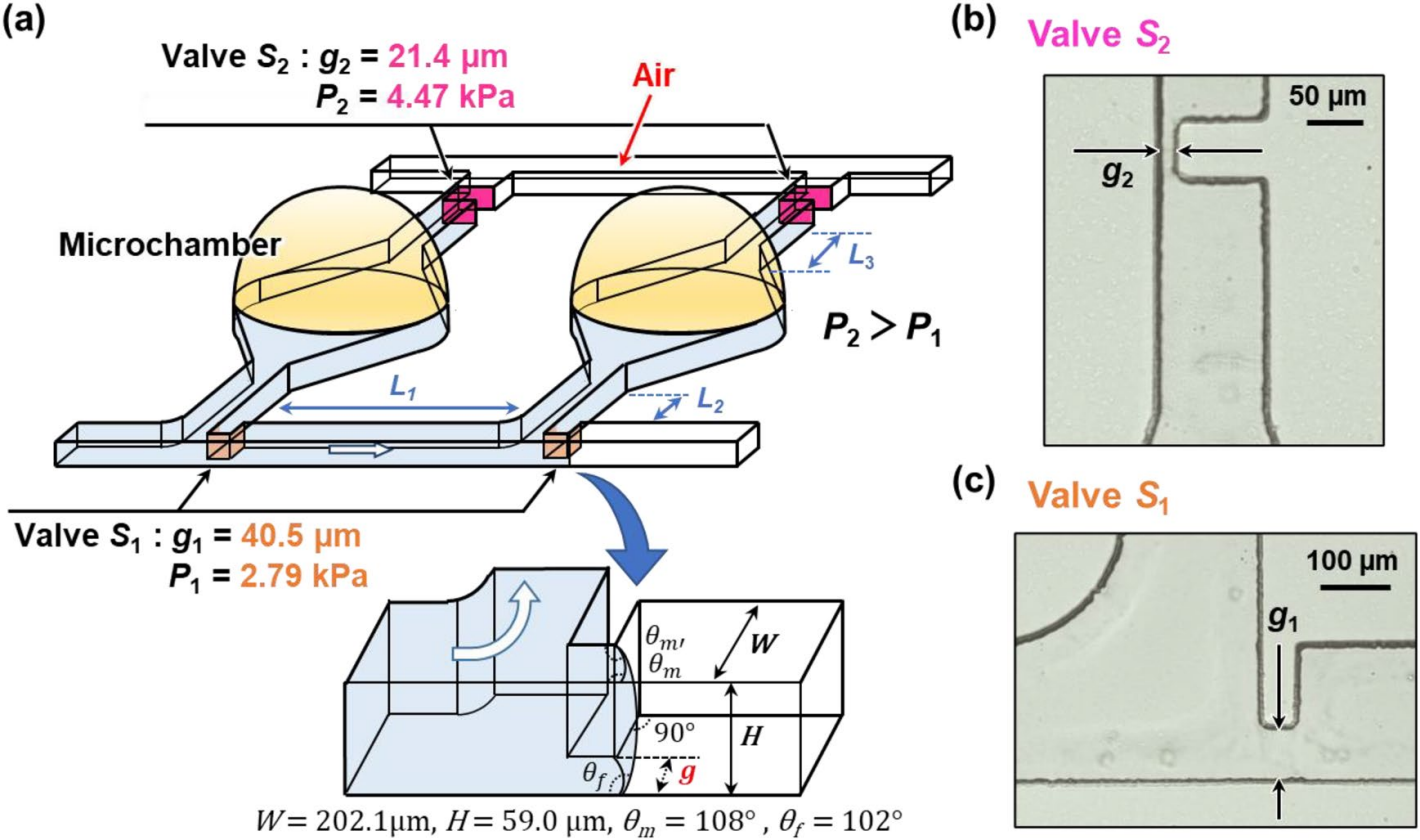
Schematic representation of a method of sequential liquid dispensing into multiple reaction microchambers. (**a**) Detailed design of microchambers with a pair of single-faced stop valves with different burst pressures. (**b**) Optical microscopy image of the permanent stop valve *S*_2_. (**c**) Optical microscopy image of the temporary stop valve *S*_1_.

In our previous study^37^, we introduced a method of sequential liquid dispensing into multiple reaction microchambers by controlling the burst pressures in a pair of passive stop valves integrated in each microchamber (Fig. 2). In brief, the dispensing procedure was as follows: first, the flow of liquid stops after it reaches the passive stop valve *S*_1_ (designed with burst pressure *P*_1_), which acts as a temporary stop valve, and is redirected toward the microchamber. After the microchamber is filled with liquid, the flow of liquid is stopped at the passive stop valve *S*_2_ (designed with burst pressure *P*_2_), which acts as a permanent stop valve, and then, the liquid flows toward the second microchamber by passing through valve *S*_1_ because *P*_1_ < *P*_2_. Valve *S*_2_ also helps exhausting the air in the microchambers. All microchambers can be sequentially filled with liquid by repeating this process. In the dispensing process, the possible number of microchambers dispensed and the maximal allowable flow rate can be theoretically designed by the sequential liquid dispensing theory that we suggested and that is described by the following equation:

1 $$\begin{array}{*{20}c} {P_{2} > P_{1} + \left( {m - 1} \right)\Delta P\left( {L_{1} } \right) + \Delta P\left( {L_{2} } \right) + \Delta P\left( {L_{3} } \right)} \\ \end{array}$$

where Δ*P*(*L*_1_), Δ*P*(*L*_2_), and Δ*P*(*L*_3_) are the pressure differences required to flow a liquid in microchannels with characteristic lengths *L*_1_, *L*_2_, and *L*_3_, respectively, and *m* is the number of microchambers that have been fully filled. Definitions of *L*_1_, *L*_2_, and *L*_3_ are given in the next section. The flow resistance inside the microchamber is not considered because the microchamber is considerably larger in space than the microchannels. The theoretical pressure drop Δ*P*(*L*) in a rectangular microchannel is given by the following equation^39^:

2 $$\begin{array}{*{20}c} {\Delta P\left( L \right) = Q \times \frac{12\eta L}{{H^{3} W}}\left( {1 - 0.630\frac{H}{W}} \right)^{ - 1} } \\ \end{array}$$

where *W*, *H*, and *L* are microchannel width, height, and length, respectively, *η* is the dynamic viscosity of the liquid (*η* = 1 mPa·s for water), and *Q* is the volumetric flow rate.

According to the dispensing theory (Eq. 1), to increase the flow rate for the same possible number of microchambers dispensed, it is required to reduce the microchannel length, in particular *L*_1_, decrease the burst pressure *P*_1_ of the temporary stop valve *S*_1_, and increase the burst pressure *P*_2_ of the permanent stop valve *S*_2_, thus reducing the time required for dispensing liquid into the microchambers. The theoretical burst pressure, *P*(*g*) (Pa), of valve *S*_1_ is described as follows^37^:

3 $$\begin{array}{*{20}c} {P\left( g \right) = - \gamma \left( {\frac{{\cos \left( {\min \left( {\theta_{m} + \beta ,180^\circ } \right)} \right) + \cos \theta_{m} }}{{g + r\left( {1 - \cos \beta } \right)}} + \frac{{\cos \theta_{m} + \cos \theta_{f} }}{H}} \right)} \\ \end{array}$$

where *g* is the gap between the vertical sidewall of the microchannel and the convex structure embedded on the opposite sidewall (hereinafter referred to as a “single-faced stop valve”), *H* is the height of the microchannel, *θ*_m_ is the water contact angle for the top and sidewall surfaces of the microchannel at a narrow gap (*θ*_m_ = 108° for PDMS), *θ*_f_ is the water contact angle for the bottom surface of the microchannel (*θ*_f_ = 102° for the silicone-based adhesive double-sided tape), and *γ* is the surface tension of the liquid (*γ* = 0.073 N/m for water). When the corner radius *r* at the back edge of the convex structure is not negligible, the denominator *g* + *r* (1 ‒ cos *β*) in the first term is the redefined gap distance between a certain position on the corner at an angle *β* and the vertical sidewall of the microchannel, where *β* is the angle between the direction perpendicular to the longitudinal direction of the microchannel and the position of the liquid–air meniscus corresponding to being pinned at the corner of the convex structure. In this study, we investigated newly designed valve configurations for the permanent stop valve *S*_2_ to improve the pressure resistance performance. We analyzed the theoretical and experimental performance of sequential liquid dispensing into an array of microchambers by implementing a double-faced stop valve or air plug-in valve (the designs of the two valves are described in detail in the next section) and compared it with that of the single-faced stop valve reported in our previous study^37^.

### Operating procedure for the multiplexed LAMP assay in the microfluidic device

The aim of this study was to introduce a rapid and easy sample-to-answer platform for the multiplexed detection of multiple allergens in processed foods in a single operation. We explored the possibility of the simultaneous detection of three specific DNA targets from wheat, buckwheat, and peanut using our newly designed microfluidic diagnostic device with 10 microchambers arranged in a circle. The operating procedure for the multiplexed colorimetric LAMP assay in the microfluidic diagnostic device for the simultaneous genetic detection of the three allergens is as follows (Supplementary Fig. S1): first, 0.5 µL of three specific primer sets designed to amplify the targeted plant allergen genes (No. 2 and 7 for wheat; No. 3 and 8 for buckwheat; and No. 4 and 9 for peanut) and 0.5 µL of universal primer sets to identify all plant species as a positive control (No. 5 and 10) were pre-spotted and dried in each reaction chamber (volume ≈ 3 µL) on a hot plate at 80 °C for 3 min. Subsequently, both the microchambers and the microchannels on the PDMS surface were sealed with a PVC sheet using a silicone-based adhesive double-sided tape. After dispensing a mixture of DNA sample and LAMP reagents into the multiple reaction chambers at a flow rate of 30 µL/min using the syringe pump, the inlet and outlet ports were sealed with a silicone-based adhesive double-sided tape along with a polyethylene terephthalate release liner on one side. Here, the release liner is laminated on both sides to cover the adhesive layers of the double-sided tape. The microfluidic device was further mechanically clipped between two glass plates (S9213; Matsunami Glass, Osaka, Japan) placed on both sides of the device to prevent leakage at the interfaces between the PVC sheet, PDMS, and polyethylene terephthalate release liner. To avoid deformation of the device by the mechanical clipping, the PDMS device was sandwiched between two additional glass plates. Finally, the device was immersed in a hot water bath (TB-2N; AS ONE, Osaka, Japan) to amplify the target DNA through the LAMP reaction under isothermal conditions at 60 °C for 60 min.

HNB (FUJIFILM Wako Pure Chemical, Osaka, Japan) was used as an indicator for the visible colorimetric detection of the LAMP reaction and for image analysis for endpoint color quantification. In our previous study^37^, we developed a method to quantitatively assess color differences between a positive reaction (sky blue) and a negative reaction (violet) after the LAMP assays, based on a representation of the CIE L*a*b* color space (CIELAB). In the CIELAB color space, L* indicates lightness, and a* and b* are chromaticity coordinates. In brief, first, the RGB values for each microchamber were quantified using ImageJ (version 1.52a, National Institutes of Health, Bethesda, MD, USA) from photographs taken with a smartphone camera and subsequently converted to *XYZ* tristimulus values in the CIE *XYZ* color space. The color parameter values L*, a*, and b* were calculated using the *XYZ* values. This calculation method has been previously thoroughly described^40^. HNB was added to the mixture of DNA sample and LAMP reagents in each reaction chamber at final concentration of 150 µM. The Loopamp DNA Amplification Kit (Eiken Chemical, Tokyo, Japan), including a 2 × reaction mixture and a thermostable *Bst* DNA polymerase, was employed to perform the LAMP reactions. DNA was extracted from wheat, buckwheat, peanut, and tea plant (*Camellia sinensis*; used as a negative control) using a TaKaRa NucleoSpin Plant II kit (Takara Bio, Shiga, Japan) and was quantified using a NanoDrop 1000 spectrophotometer (Thermo Fisher Scientific, Waltham, MA, USA). Four plant samples including *T. aestivum* (voucher no. JU2021TA10), *F. esculentum* (voucher no. JU2021FE11), *A. hypogaea* (voucher no. JU2021AH12), and *C. sinensis* (voucher no. JU2021CS13) were obtained from the botanical garden of Josai University. All specimens were deposited at the botanical garden of Josai University.

## Results and discussion

### Reproducibility of microchamber structures constructed based on wax reflow

**Figure 3:**
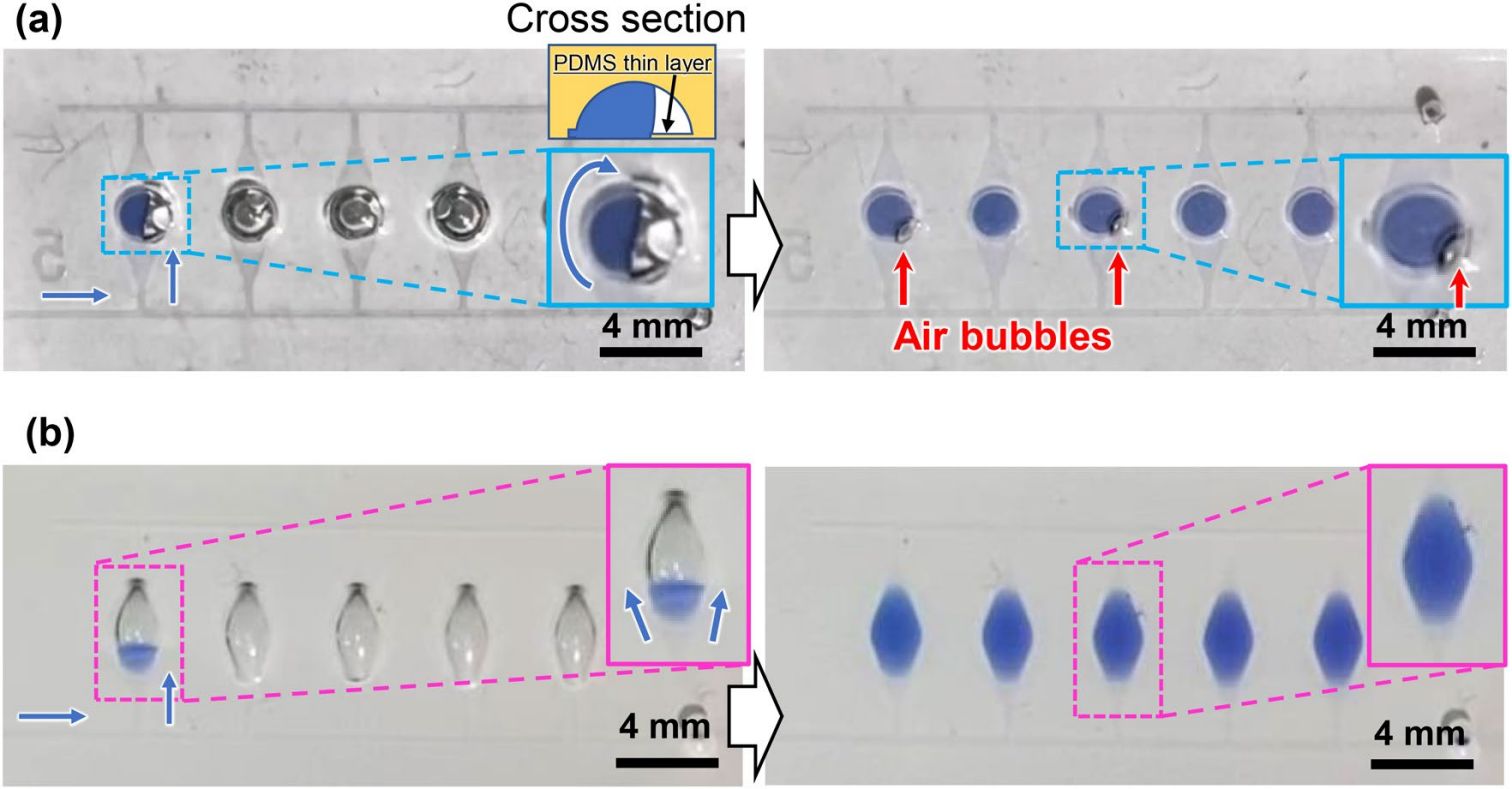
Comparison of liquid dispensing behaviors between PDMS microchambers fabricated by employing (**a**) hemispherical polymer beads and (**b**) the wax reflow process. The former occasionally exhibited unexpected air bubbles trapped in the microchambers, whereas the latter did not.

In our previous studies^35–37^, we used hemispherical polymer beads (2 mm in diameter; SAYAKOBO, Yokohama, Japan) to create deep localized microchamber structures instead of the wax reflow process (Supplementary Fig. S2). The polymer beads were manually glued onto each SU-8 chamber pattern as a mold using an epoxy adhesive (Araldite; Huntsman Japan, Kobe, Japan) at room temperature for 12 h. This fabrication process led to low reproducibility of the microchambers, i.e., the low controllability of the adhesive layer thickness and excess adhesive being squeezed out at the bonding interface decreased the replication accuracy, particularly at the intersection of the microchannel and the entrance of the microchamber. Such process failures sometimes led to unexpected air bubbles being trapped in the microchamber (Fig. 3a). This unfavorable result was most likely caused by a non-uniform flow on a step or around an obstacle at the entrance of the microchamber. The probability of sample dispensing without air bubbles was 87.0% in experiments with 20 devices and a total of 100 chambers. By implementing the thermal reflow process using wax, the probability of dispensing without air bubbles was markedly improved up to 100% in experiments with 20 devices and a total of 100 chambers (Fig. 3b).

### Sequential liquid dispensing using a double-faced stop valve

**Figure 4:**
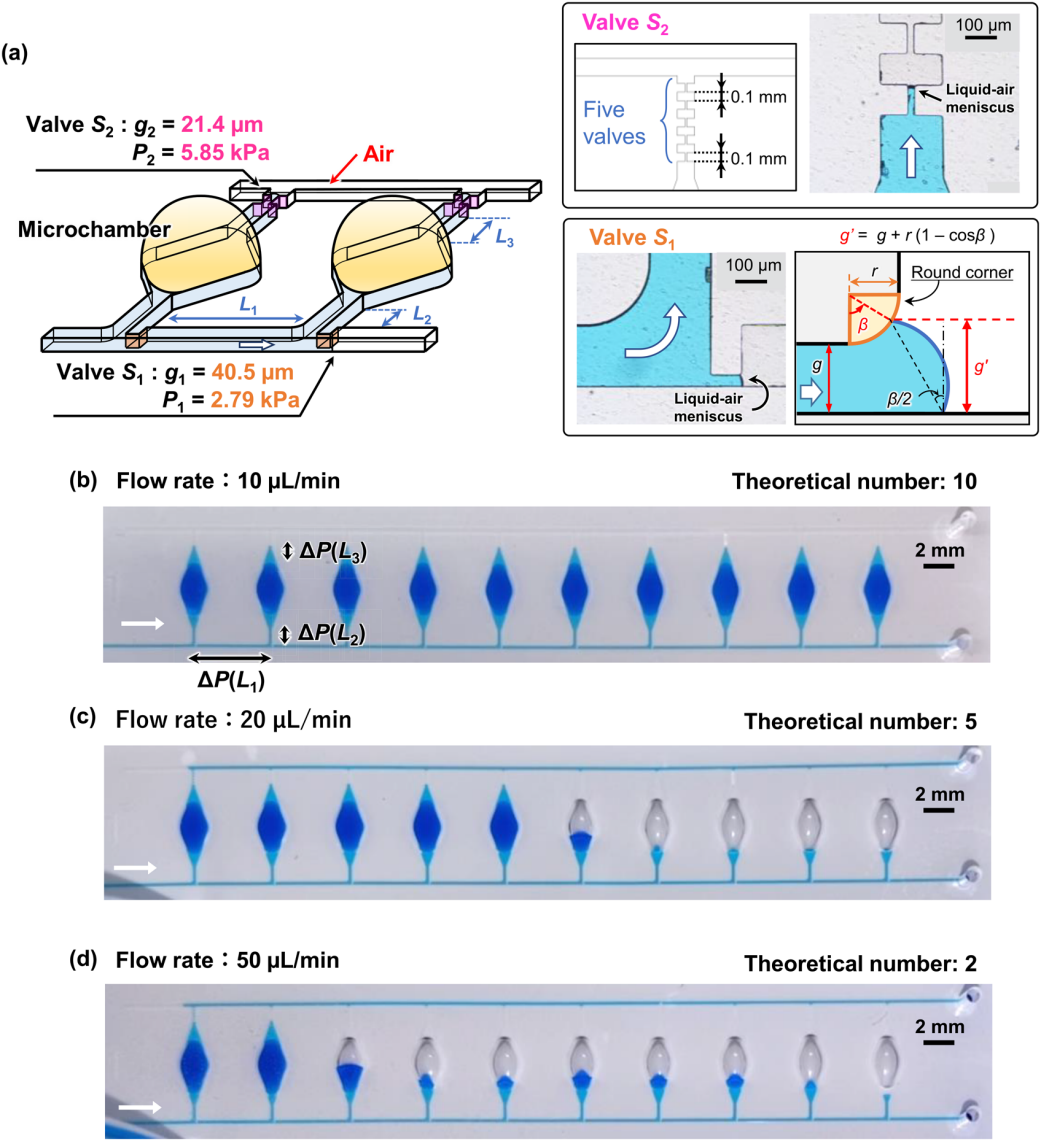
Experimental results showing the effect of the flow rate on the performance of sequential liquid dispensing in an array of 10 microchambers with double-faced stop valves. (**a**) Detailed design of the reaction microchambers with a pair of passive stop valves including a single-faced stop valve as a temporary stop valve *S*_1_ and a double-faced stop valve as a permanent stop valve *S*_2_. The microchambers were sequentially filled with blue-colored water at a flow rate of (**b**) 10, (**c**) 20, or (**d**) 50 µL/min.

In this study, we improved the pressure resistance performance of valve *S*_2_ by changing its geometrical configuration. As shown in Fig. 4a, the gap distance of valve *S*_2_ is determined by two convex structures facing each other embedded on both sidewalls of the microchannel (referred to as a capillary stop valve^41^). In our devices, the valve structure of *S*_2_ is hereinafter referred to as a double-faced stop valve, whereas the *S*_1_ valve is a single-faced stop valve. The theoretical burst pressure of the double-faced stop valve can be derived as follows:

4 $$\begin{array}{*{20}c} {P\left( g \right) = - \gamma \left( {\frac{{2\cos \left( {\min \left( {\theta_{m} + \beta ,180^\circ } \right)} \right)}}{{g + 2r\left( {1 - \cos \beta } \right)}} + \frac{{\cos \theta_{m} + \cos \theta_{f} }}{H}} \right)} \\ \end{array}$$

The gap distance of the double-faced stop valve *S*_2_ was experimentally measured to be *g*_2_ = 21.4 μm (Fig. 4), resulting in a theoretical burst pressure *P*_2_ of 5.85 kPa for *r*_2_ = 6.4 µm in a microchannel (*W* = 202.1 µm and *H* = 59.0 µm). The theoretical burst pressure of the single-faced stop valve with the same gap distance (*g*_2_ = 21.4 μm) was calculated to be *P*_2_ = 4.47 kPa. Thus, the burst pressure can be increased by 1.3 times compared to that of the single-faced stop valve by implementing the double-faced stop valve for *S*_2_. It should be noted that five pairs of *S*_2_ were arranged in series (Fig. 4a) because we assumed that the gap distance (*g*_2_) and/or corner radius (*r*_2_) of one or more valves would not be the design value because of unexpected process failures in soft lithography. Thus, the one of the five valves that has the highest burst pressure (*P*_2_) determines the actual valve performance limit in microfluidic devices. For comparison, the gap distance *g*_1_ of 40.5 μm for the single-faced stop valve *S*_1_ resulted in a theoretical burst pressure *P*_1_ of 2.79 kPa. The microchannel lengths *L*_1_, *L*_2_, and *L*_3_ were set as 5.0 mm, 1.0 mm, 0.2 mm, as shown in Fig. 4a.

Figure 4b shows that an array of 10 microchambers was filled completely with water colored with blue food color (0.1% w/v) when we used the pressure-driven micropump at a flow rate of 10 µL/min. As expected, the possible number of microchambers dispensed when using the double-faced stop valve *S*_2_ was increased by 1.7 times as compared to the six microchambers that could be achieved at the same flow rate with the single-faced stop valve *S*_2_ used previously^37^. However, as predicted by the theoretical dispensing model, the possible number of microchambers dispensed decreased to five and two when the flow rate was increased to 20 µL/min (Fig. 4c and Video S1) or 50 µL/min (Fig. 4d), respectively.

### Sequential sample dispensing using an air plug-in valve

According to the dispensing theory described in Eqs. (1–4), the maximal allowable flow rate is limited by an increase in the flow resistance Δ*P*(*L*_1_), which increases in proportion to not only the flow rate, but also the number of microchambers filled. When using the geometric dimensions of the microchannels and a pair of passive stop valves as designed in this study, the maximal allowable flow rate for dispensing 10 microchambers was limited to approximately 10 µL/min. It should be noted that a further reduction of the gap distance (*g*_2_ = 20 µm as a design value) of the permanent stop valve *S*_2_ would improve the pressure resistance (*P*_2_), but would negatively affect the patterning accuracy of the SU-8 mold by photolithography.

**Figure 5:**
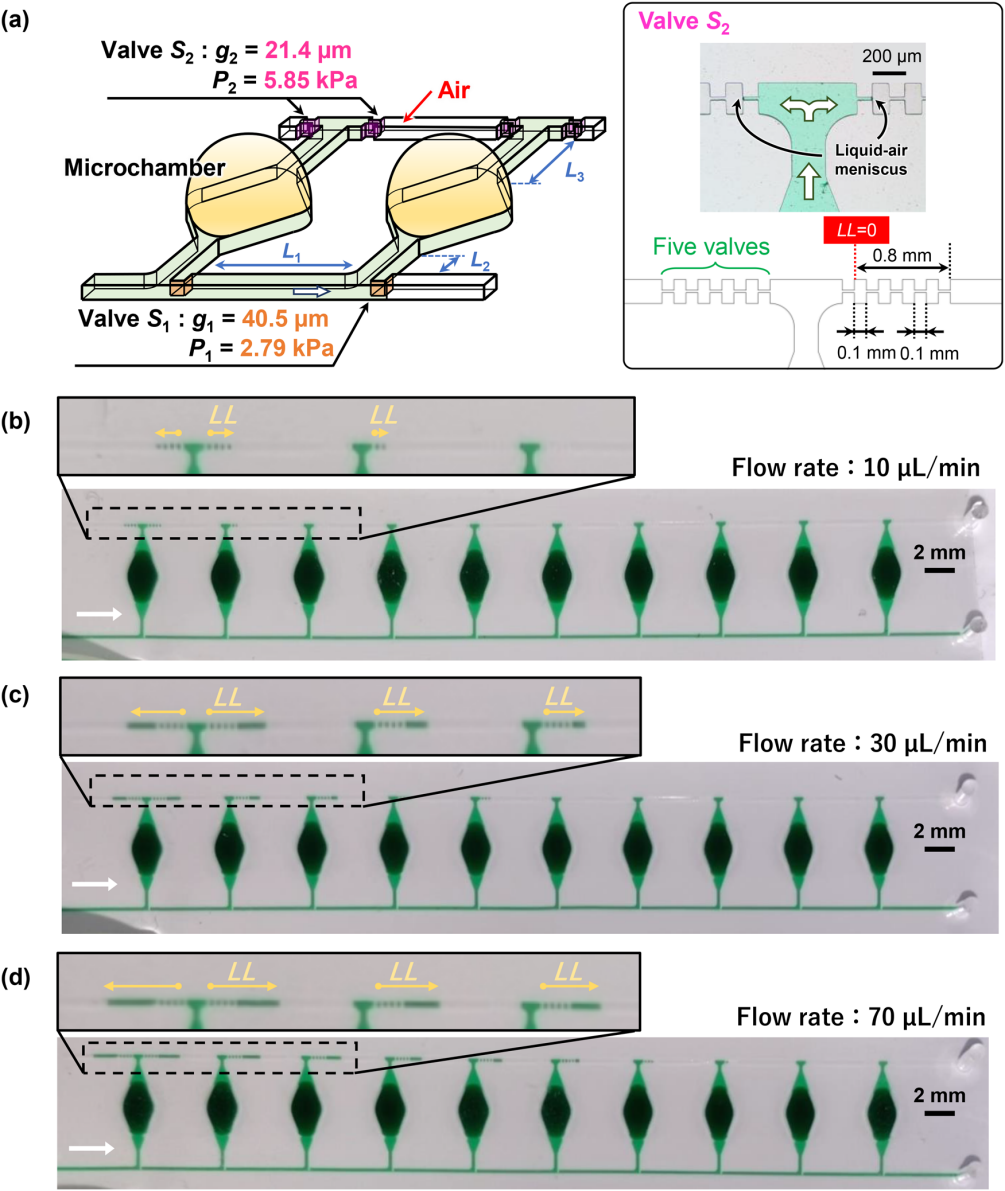
Experimental results showing the effect of the flow rate on the performance of sequential liquid dispensing in an array of 10 microchambers with air plug-in valves. (**a**) Detailed design of the reaction microchambers with the air plug-in valve as a permanent stop valve S_2_, consisting of a set of two double-faced stop valves facing each other in the air exhaust microchannel. The microchambers were sequentially filled with green-colored water at a flow rate of (**b**) 10, (**c**) 30, or (**d**) 70 µL/min. *LL* in the photographs stands for leakage length.

Therefore, we propose a newly designed valve configuration for the permanent stop valve, which we termed an “air plug-in valve.” As shown in Fig. 5a, a set of two double-faced stop valves *S*_2_ was placed downstream and upstream in the upper microchannel (hereinafter referred to as the air exhaust microchannel) for exhausting the air in the microchambers. Five pairs of *S*_2_ were arranged in series to avoid the risk of unexpected failures in the fabrication process as mentioned in the previous section. The most distinctive feature of the air plug-in valve proposed here is that air is trapped in the part of the air exhaust microchannel connecting two *S*_2_ valves of neighboring microchambers when the next microchamber is completely filled with liquid (Fig. 5a). For example, both *S*_2_ valves of the first microchamber are subjected to the total pressure given on the right side of Eq. (5a) while the second microchamber is being filled with liquid. This condition corresponds to the case where *m* = 1 in Eq. (1), and the pressure applied to the *S*_2_ valves reaches the maximum right before the liquid reaches the *S*_2_ valves of the second microchamber. However, once the air trapping between the *S*_2_ valves of the first and second microchambers is completed, the pressure applied to the *S*_2_ valves of the first microchamber decreases to the pressure given on the right side of Eq. (5b) because the liquids reaching the two valves push against each other through the air plug, resulting in the offsetting of the applied pressure.

5a $$\begin{array}{*{20}c} {P_{2} > P_{1} + \Delta P\left( {L_{1} } \right) + \Delta P\left( {L_{2} } \right) + \Delta P\left( {L_{3} } \right)} \\ \end{array}$$

5b $$\begin{array}{*{20}c} {P_{2} > \Delta P\left( {L_{1} } \right)} \\ \end{array}$$

It can be concluded that the burst pressure *P*_2_ of the permanent stop valves *S*_2_ should be designed only to satisfy the constraint given by Eq. (5a), i.e., they are required to have flow resistance only until the liquid has been fully dispensed into the next microchamber. This also means that the limitation to the possible number of microchambers dispensed has been removed by implementing the air plug-in valve for *S*_2_.

Figure 5b–d shows that all 10 microchambers were sequentially filled with water containing green food color (0.1% w/v) at flow rates of 10, 30, and 70 μL/min (Video S2). Thus, the air plug-in valve allowed successful dispensing into all 10 microchambers at flow rates below 70 μL/min. According to the constraint given by Eq. (5a), the theoretical maximal allowable flow rate is limited to approximately 70 µL/min for dispensing 10 microchambers given the geometric dimensions of the microchannels and the pair of passive stop valves in the design used in this study. Thus, the experimental results satisfactorily agreed with the theoretically predicted maximal allowable flow rate. The allowable flow rate achieved with the implementation of the air plug-in valve was seven times higher than that obtained with the previous valve arrangement using double-faced stop valves and 14 times higher than that obtained with the single-faced stop valves used in our previous study (the maximal allowable flow rate was 5 μL/min for 10 microchambers)^37^. Notably, the dramatic improvement in the pressure resistance of the permanent stop valves allowed us to introduce the liquid manually into the microfluidic device (Video S3; estimated mean flow rate: ~ 40 µL/min).

### Unexpected behavior of the air plug-in valve

**Figure 6:**
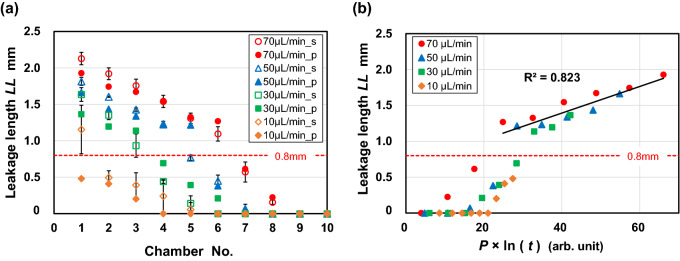
Experimental analysis of leakage length (*LL*). (**a**) *LL* values at the air plug-in valve downstream of each microchamber at flow rates of 10–70 µL/min. Open and solid symbols in the graph represent data for PDMS microfluidic devices sealed with silicone-based adhesive double-sided tape and covalently bonded to a thin PDMS layer, respectively. (**b**) The data for the PDMS device that was covalently bonded to a thin PDMS layer coated on a 4-inch glass wafer were replotted as a function of the product of the internal pressure (*P*) applied to each air plug and the natural logarithm of time (ln(*t*)).

Unexpectedly, we observed that the liquid–air meniscus, which had previously been pinned by the air plug-in valve, gradually leaked downstream and, at the first microchamber, both upstream and downstream, as the dispensing proceeded (yellow arrows in Fig. 5b–d). This unexpected valve leakage behavior was most likely due to the gas permeability of PDMS^42^. The leakage length (*LL*) seemed to increase not only with an increasingly upstream position of the valve in the device, but also with an increase in flow rate. Figure 6a shows *LL* at the air plug-in valve downstream of each microchamber at flow rates of 10–70 µL/min. The values of *LL* were estimated right after the 10th microchamber was completely filled. *LL* gradually decreased as the valves were located further downstream until it reached 0.8 mm, after which it decreased with a larger negative slope. Five pairs of *S*_2_ were arranged in series in the air exhaust microchannel, resulting in a total length of 0.8 mm between the back edge of the first valve convex structure (set to the zero position of *LL*) and the back edge of the fifth valve (Fig. 5a). The transition region near *LL* = 0.8 mm was most likely due to the geometric differences in the microchannel, i.e., periodic fluctuations in the microchannel width due to the facing convex structures embedded on both sidewalls of the microchannel.

To investigate the effect of air leakage at the interface of the PDMS device and the silicone-based adhesive double-sided tape, we prepared a modified PDMS device that was covalently bonded to a thin PDMS layer coated on a 4-inch glass wafer by air plasma surface treatment. The *LL* values measured for the modified PDMS device are also plotted in Fig. 6a. It can be seen that there was no significant difference between two types of devices. Thus, negligible air leakage occurred at the interface between the PDMS device and adhesive tape. In addition, although all air plug-in valves *S*_2_ were subjected to an applied pressure described in Eq. (5b) right after the air trapping between adjacent valves was completed, the air plug trapped between the first and second microchambers exhibited a larger internal pressure than any other air plugs located downstream (Fig. S3), i.e., the internal pressure increased with an increase in the number of microchambers that had been dispensed, according to Eq. (1). Therefore, *LL* can be considered to increase not only at air-plug in valves located further upstream, but also at a higher flow rate because of an increase in flow resistance that can be estimated by Eq. (2).

In Fig. 6b, the data for the PDMS device covalently bonded to a thin PDMS layer coated on a 4-inch glass wafer are replotted as a function of the product of the internal pressure (*P*) applied to each air plug and the natural logarithm of time (ln(*t*)). *P* was theoretically calculated according to Eq. (1), and *t* was experimentally determined as the total time elapsed between a certain air plug being completed and all 10 microchambers being completely filled with water. The data are listed in Table S1 in the supplementary information. As shown in the graph, under the conditions of *LL* ≥  0.8 mm, *LL* is expressed regardless of the flow rate and valve position as follows:

6 $$\begin{array}{*{20}c} {LL \propto P \times \ln \left( t \right)} \\ \end{array}$$

where the coefficient of determination (R^2^) was estimated to be 0.823, indicating that the assumption presented in Eq. (6) is valid. Therefore, we can theoretically predict *LL* even during the design process of microfluidic devices. It should be noted that, when pumping stopped the flow of liquid into the microfluidic device, the inlet tubing was removed, and the leakage of liquid at the air plug-in valves was significantly reduced because the internal pressure in both the microchannel and microchambers immediately decreases to atmospheric pressure. A fundamental way to overcome the leakage problem is to replace PDMS with engineering plastics with lower gas permeability, such as polymethyl methacrylate, polycarbonate, or cyclo-olefin polymer.

### Multiplexed LAMP assay for the detection of food allergens

**Figure 7:**
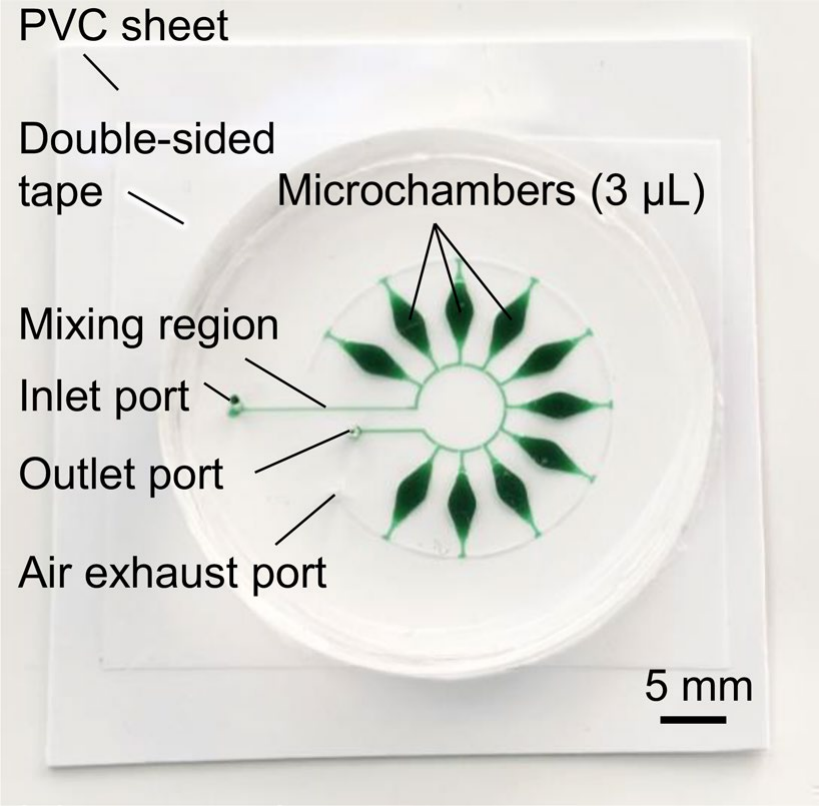
Optimally designed PDMS microfluidic device with 10 reaction microchambers (~ 3 µL each) arranged in a circle and compactly housed within the 20-mm outermost diameter formed by the air exhaust microchannel, employed for the multiplexed genetic detection of food allergens. All microchambers were sequentially filled with green-colored water at a flow rate of 30 µL/min.

We explored the possibility of the rapid, simultaneous detection of multiple food allergenic substances i.e., specific DNA targets from wheat (*T. aestivum*), buckwheat (*F. esculentum*), and peanut (*A. hypogaea*), by the colorimetric LAMP method using a microfluidic device with a circular arrangement. With the aim of providing a microfluidic diagnostic device for the simultaneous genetic detection of seven allergens in processed foods, we compactly housed 10 microchambers in a circle instead of using a single row format. As shown in Fig. 7, in a device with a circular arrangement, green-colored water (0.1% w/v) could be successfully sequentially dispensed into all microchambers at a flow rate of 30 µL/min (Video S4). Our sequential liquid dispensing method provides substantial flexibility in the design of microfluidic devices, thus allowing the geometric design of 10 microchambers arranged in a circle and compactly housed within the 20-mm outermost diameter formed by the air exhaust microchannel. The geometric dimensions of this type of device were designed as *L*_1_ = 1.52 mm, *L*_2_ = 0.80 mm, and *L*_3_ = 0.89 mm. The theoretical maximal allowable flow rate for dispensing water into the 10 microchambers was estimated to be below 160 µL/min, the gap distances were *g*_1_ = 40.5 μm and *g*_2_ = 21.4 μm for valves *S*_1_ and *S*_2_, respectively, the corner radius at the back edge of the convex structures was *r*_1_ = *r*_2_ = 6.4 µm, and the width and height of the microchannel were *W* = 202.1 µm and *H* = 59.0 µm, respectively.

**Figure 8:**
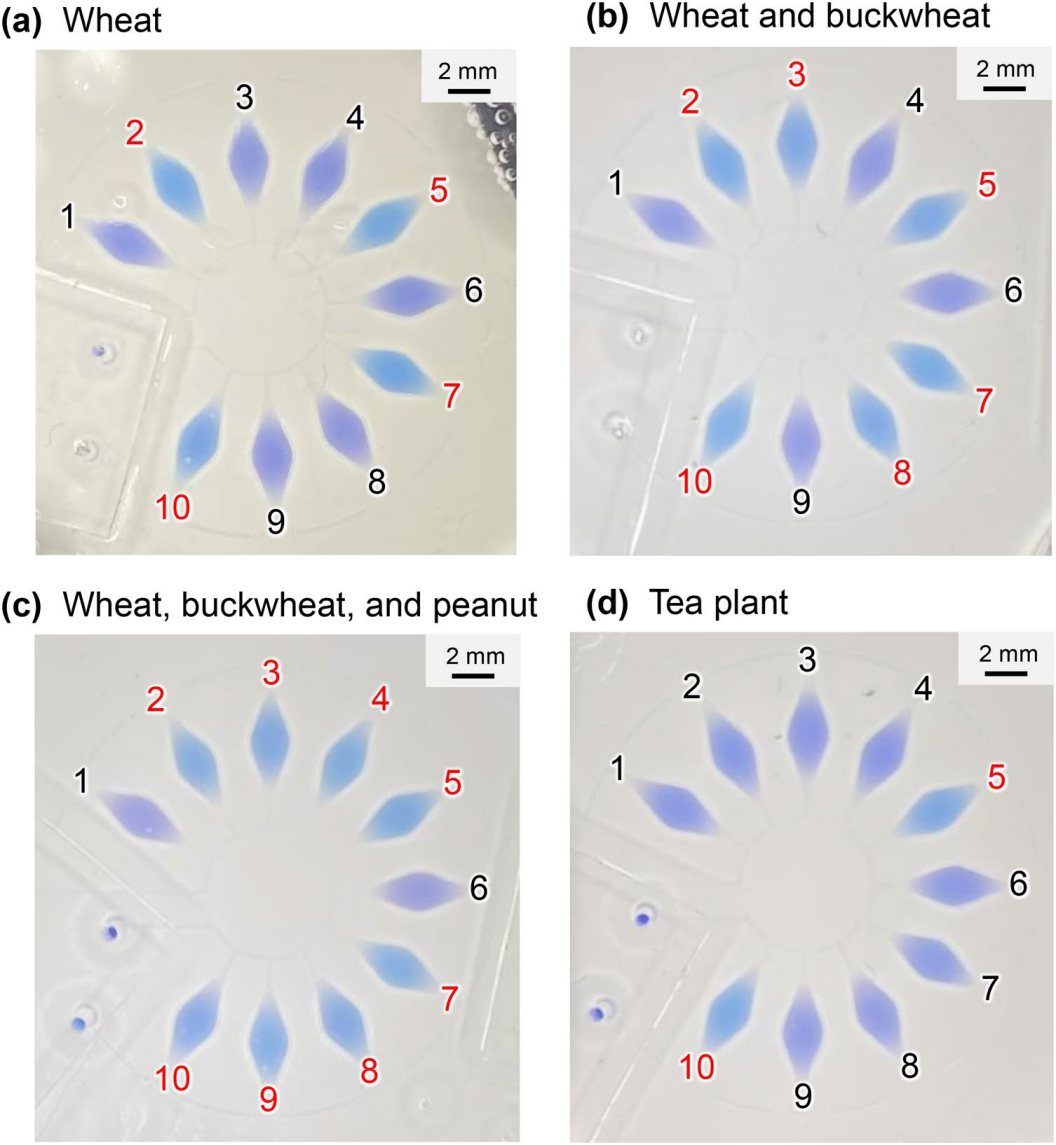
Photographs taken with a smartphone camera showing the colorimetric detection of (**a**) wheat DNA (Nos. 2 and 7), (**b**) a mixture of wheat and buckwheat DNA (Nos. 3 and 8), (**c**) a mixture of wheat, buckwheat, and peanut DNA (Nos. 4 and 9), and (**d**) tea plant DNA as a negative control, by LAMP assays run at 60 °C for 60 min. Universal primer sets to identify all plant species were pre-spotted in chamber Nos. 5 and 10 as a positive control, whereas no primers were pre-spotted in chamber Nos. 1 and 6 as a negative control.

Figure 8a shows an experimental result for the detection of wheat-specific DNA (total DNA concentration: 1.0 ng/µL) by the colorimetric LAMP method using the microfluidic device. After the wheat DNA sample mixed with the LAMP reagents was introduced into the device, the LAMP assay was conducted in a hot water bath at 60 °C for 60 min. As expected, positive reactions, manifested by a color change of HNB (150 µM) in the LAMP reaction solution from violet to sky blue, were clearly observed in chambers 2, 5, 7, and 10, without false-positive and false-negative results. LAMP assays with buckwheat DNA and peanut DNA yielded true-positive results in chambers 3 and 8 (Supplementary Fig. S4a) and 4 and 9 (Fig. S4b), respectively, as well as in chambers 5 and 10 used as a positive control.

Figure 8b shows the simultaneous detection of multiple food allergens in a mixture of wheat DNA and buckwheat DNA (total DNA concentration: 1.0 ng/µL each) by the LAMP assay; positive reactions occurred in chambers 2, 3, 5, 7, 8, and 10, without any cross-contamination. Multiplexed LAMP assays with other combinations of the DNA samples, i.e., mixtures of wheat DNA and peanut DNA (Fig. S4c) and buckwheat DNA and peanut DNA (Fig. S4d), also yielded true test results. Similarly, a mixture of all three DNA samples (1.0 ng/µL each) yielded positive reactions in all chambers except chambers 1 and 6, used as a negative control (Fig. 8c). In contrast, when DNA extracted from a tea plant (1.0 ng/µL) was introduced into the device, a color change from violet to sky blue was observed only in the positive control chambers (Nos. 5 and 10), without false-positive reactions in the other chambers (Fig. 8d).

**Figure 9:**
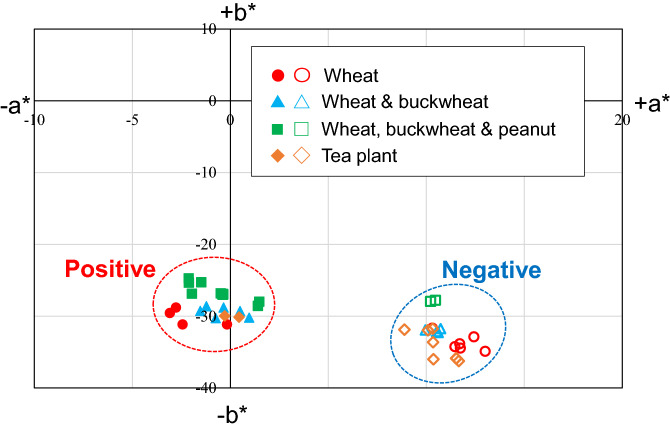
Color distributions for positive (sky blue) and negative (violet) LAMP reactions (shown in Fig. 8) run for the detection of three food allergens (wheat, buckwheat, and peanut) and tea plant as a negative control, plotted in the a*-b* chromatic plane of the CIELAB color space. LAMP reactions were run at 60 °C for 60 min.

Figure 9 shows the distributions of color differences plotted in the a* − b* chromatic plane of the CIELAB space from the LAMP results presented in Fig. 8 (similarly, the data presented in Fig. S4 are plotted in Supplementary Fig. S5). The result revealed that the colors of the positive and negative groups were clearly separated after the LAMP assays were run for 60 min, for any combination of the three food allergens and tea plant used as a negative control.

## Conclusions

With the aim of improving the performance of PDMS-based microfluidic devices employed for the rapid and easy-to-use multiplexed genetic diagnostics, we proposed a newly designed valve configuration, termed an air plug-in valve. The pressure resistance performance of the air plug-in valve was remarkably enhanced after the air trapping between facing valves was completed. By replacing the previous valve arrangement using double-faced stop valves with the air plug-in valves, which was used as a permanent stop valve, the maximal allowable flow rate significantly increased by up to 14 times (~ 70 µL/min) for sequentially dispensing a liquid to fill an array of 10 reaction microchambers. Further, the limitation to the possible number of microchambers dispensed was removed by implementing the air plug-in valve. We demonstrated that the fabricated microfluidic device enables the simultaneous detection of three plant food allergens (wheat, buckwheat, and peanut) by colorimetric LAMP assays run at 60 °C for 60 min in a single operation, without cross-contamination between the reaction microchambers. It should be noted that the diagnostic device could be designed with a geometrical configuration of 10 reaction microchambers arranged in a circle and compactly housed within a 20-mm outermost diameter because our sequential liquid dispensing method provides substantial flexibility in the design of microfluidic devices.

In future studies, to simplify the operating procedure, we will further develop not only a method to pre-store the LAMP reagents in the microfluidic device, but also a pumpless microfluidic device based on a combination of centrifugal pumping and capillary valving, without the need for an external pumping system (i.e., a syringe or pressure pump). The final goal is to develop a rapid and easy sample-to-answer diagnostic platform that allows the simultaneous detection of not only all seven allergenic ingredients that must be labeled under Japanese law, i.e., wheat, buckwheat, peanut, egg, milk, shrimp, and crab, but also foodborne pathogens (e.g., *Salmonella enterica*, *S. aureus*, *Campylobacter jejuni*, and *E. coli* O157:H7) using our microfluidic device, to ensure food safety and security. In principle, it is possible to flexibly customize the type of nucleic acid target of interest (DNA/RNA); thus, this highly versatile technology can be applied to the on-site multiplexed genetic detection of not only allergens, but also infectious diseases caused by various pathogens (viruses, bacteria, fungi, and parasites), poisonous plants, and illegal drugs, without a need for laborious and multiple operations.

## Supplementary Information

Supplementary Information.

Supplementary Video S1.

Supplementary Video S2.

Supplementary Video S3.

Supplementary Video S4.

## Data Availability

All data relevant to the study are included in the article or uploaded as supplementary information.
