# Supplementary material for: A microfluidic diagnostic device with air plug-in valves for the simultaneous genetic detection of various food allergens
Source: Sci Rep. 2022 Jul 27;12:12852. doi: 10.1038/s41598-022-16945-2 (PMC9329328; doi:10.1038/s41598-022-16945-2)
Supplement: Supplementary file 1 — Supplementary Information. [file 41598_2022_16945_MOESM1_ESM.docx]

Supplementary Information

**A microfluidic diagnostic device with air plug-in valves for the simultaneous genetic detection of various food allergens**

Daigo Natsuhara^1,*^, Sae Misawa^2^, Ryogo Saito^1^, Koki Shirai^1^, Shunya Okamoto^1^, Moeto Nagai^1^, Masashi Kitamura^2^ & Takayuki Shibata^1,*^

^1^ Department of Mechanical Engineering, Toyohashi University of Technology, Toyohashi, Aichi 441-8580, Japan.

^2^ Faculty of Pharmacy and Pharmaceutical Sciences, Josai University, Sakado, Saitama 350-0295, Japan.

*Corresponding authors:

Daigo Natsuhara (d-natsuhara@mems.me.tut.ac.jp), Takayuki Shibata (shibata@me.tut.ac.jp)

**Figure S1.** Operating procedure for the multiplexed LAMP assay employed in the microfluidic diagnostic devices for simultaneous detection of food allergens.


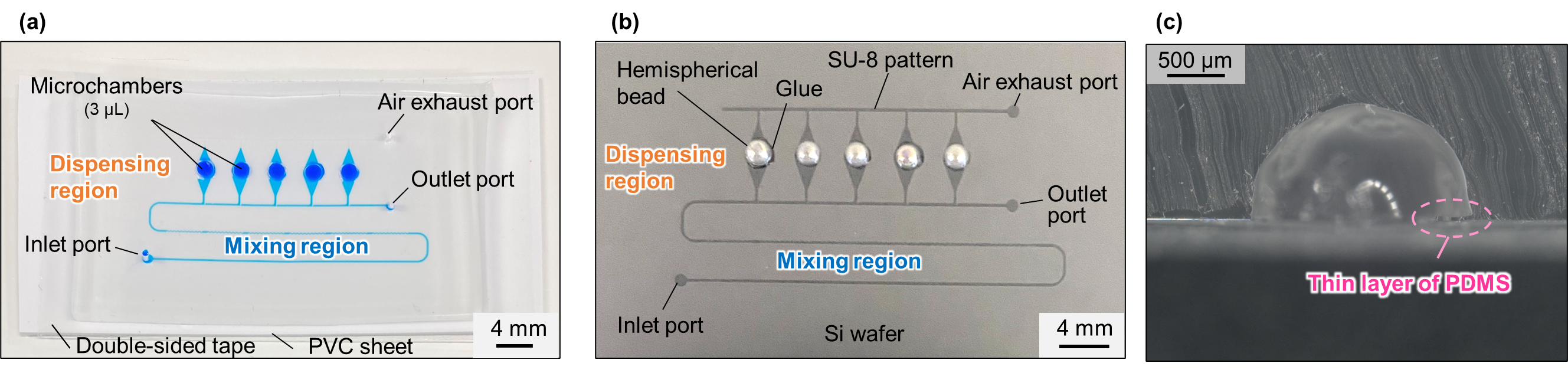


**Figure S2.** Schematic representation of the microfluidic diagnostic device fabricated through a modified soft lithography process using hemispherical polymer beads to create deep localized microchamber structures. (**a**) A fabricated PDMS microfluidic device consisting of an array of five reaction microchambers (~3 µL each) was filled with blue-colored water. (**b**) Hemispherical polymer beads were glued manually in the center on the top surface of each SU-8 chamber pattern to create a master mold for the replication of the PDMS microfluidic devices. (**c**) Cross-section of a PDMS microchamber fabricated from a hemispherical bead. An unexpected thin layer of PDMS was overhanging into the microchamber area because of poor controllability of the uniformity of the adhesive layer thickness.


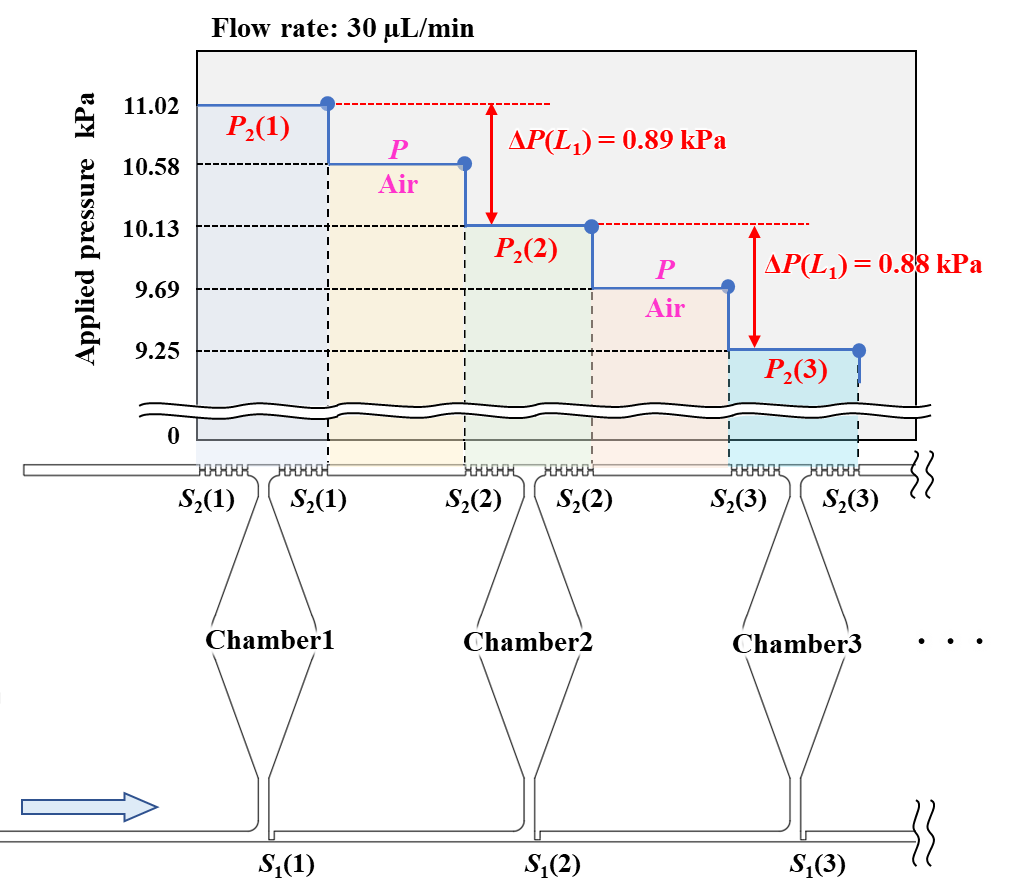


**Figure S3.** Schematic diagram of pressure distribution in the air exhaust microchannel directly after the 10th microchamber was filled with water. In this figure, only the first three microchambers are illustrated.

**Table S1.** The data for the PDMS device covalently bonded to a thin PDMS layer coated on a 4-inch glass wafer. *P*_2_ (*n*) and *P*_2_ (*n*+1) were theoretically calculated according to Eq. 1, which were the pressures applied to the right-side *S*_2_ and the left-side *S*_2_ valves of (*n*)th and (*n*+1)th microchambers, respectively, after the 10th microchamber was filled with water. *P* is the mean value, {*P*_2_ (*n*) + *P*_2_ (*n*+1)}/2, induced inside the air plug between the *S*_2_ valves facing each other in the air exhaust microchannel.

| Flow rate: 10 µL/min | | | | | | |
| --- | --- | --- | --- | --- | --- | --- |
| Chamber No.  (*n*-th) | *LL*  (mm) | *P*_2_ (*n*)  (kPa) | *P*_2_ (*n*+1)  (kPa) | *P*  (kPa) | *t*  (s) | *P* × ln (*t*) |
| 1 | 0.48 | 5.53 | 5.24 | 5.39 | 162.0 | 27.40 |
| 2 | 0.41 | 5.24 | 4.94 | 5.09 | 144.0 | 25.30 |
| 3 | 0.20 | 4.94 | 4.65 | 4.80 | 126.0 | 23.19 |
| 4 | 0 | 4.65 | 4.35 | 4.50 | 108.0 | 21.07 |
| 5 | 0 | 4.35 | 4.06 | 4.21 | 90.0 | 18.92 |
| 6 | 0 | 4.06 | 3.76 | 3.91 | 72.0 | 16.72 |
| 7 | 0 | 3.76 | 3.47 | 3.61 | 54.0 | 14.42 |
| 8 | 0 | 3.47 | 3.17 | 3.32 | 36.0 | 11.90 |
| 9 | 0 | 3.17 | 2.79 | 2.98 | 18.0 | 8.62 |
| 10 | 0 | 2.79 | 0 | 1.40 | 0 | — |

| Flow rate: 30 µL/min | | | | | | |
| --- | --- | --- | --- | --- | --- | --- |
| Chamber No.  (*n*-th) | *LL*  (mm) | *P*_2_ (*n*)  (kPa) | *P*_2_ (*n*+1)  (kPa) | *P*  (kPa) | *t*  (s) | *P* × ln (*t*) |
| 1 | 1.37 | 11.02 | 10.13 | 10.58 | 54.0 | 42.19 |
| 2 | 1.20 | 10.13 | 9.25 | 9.69 | 48.0 | 37.51 |
| 3 | 1.14 | 9.25 | 8.36 | 8.80 | 42.0 | 32.91 |
| 4 | 0.69 | 8.36 | 7.48 | 7.92 | 36.0 | 28.38 |
| 5 | 0.39 | 7.48 | 6.59 | 7.03 | 30.0 | 23.92 |
| 6 | 0.21 | 6.59 | 5.71 | 6.15 | 24.0 | 19.54 |
| 7 | 0 | 5.71 | 4.82 | 5.26 | 18.0 | 15.21 |
| 8 | 0 | 4.82 | 3.93 | 4.38 | 12.0 | 10.88 |
| 9 | 0 | 3.93 | 2.79 | 3.36 | 6.0 | 6.02 |
| 10 | 0 | 2.79 | 0 | 1.40 | 0.0 | — |

| Flow rate: 50 µL/min | | | | | | |
| --- | --- | --- | --- | --- | --- | --- |
| Chamber No.  (*n*-th) | *LL*  (mm) | *P*_2_ (*n*)  (kPa) | *P*_2_ (*n*+1)  (kPa) | *P*  (kPa) | *t*  (s) | *P* × ln (*t*) |
| 1 | 1.66 | 16.50 | 15.03 | 15.77 | 32.4 | 54.84 |
| 2 | 1.44 | 15.03 | 13.55 | 14.29 | 28.8 | 48.02 |
| 3 | 1.34 | 13.55 | 12.08 | 12.81 | 25.2 | 41.35 |
| 4 | 1.23 | 12.08 | 10.60 | 11.34 | 21.6 | 34.84 |
| 5 | 1.22 | 10.60 | 9.12 | 9.86 | 18.0 | 28.50 |
| 6 | 0.38 | 9.12 | 7.65 | 8.39 | 14.4 | 22.37 |
| 7 | 0.07 | 7.65 | 6.17 | 6.91 | 10.8 | 16.44 |
| 8 | 0 | 6.17 | 4.70 | 5.43 | 7.2 | 10.73 |
| 9 | 0 | 4.70 | 2.79 | 3.74 | 3.6 | 4.79 |
| 10 | 0 | 2.79 | 0 | 1.40 | 0 | — |

| Flow rate: 70 µL/min | | | | | | |
| --- | --- | --- | --- | --- | --- | --- |
| Chamber No.  (*n*-th) | *LL*  (mm) | *P*_2_ (*n*)  (kPa) | *P*_2_ (*n*+1)  (kPa) | *P*  (kPa) | *t*  (s) | *P* × ln (*t*) |
| 1 | 1.93 | 21.99 | 19.92 | 20.96 | 23.4 | 66.07 |
| 2 | 1.74 | 19.92 | 17.86 | 18.89 | 20.8 | 57.33 |
| 3 | 1.67 | 17.86 | 15.79 | 16.82 | 18.2 | 48.81 |
| 4 | 1.55 | 15.79 | 13.72 | 14.76 | 15.6 | 40.54 |
| 5 | 1.33 | 13.72 | 11.66 | 12.69 | 13.0 | 32.55 |
| 6 | 1.27 | 11.66 | 9.59 | 10.62 | 10.4 | 24.88 |
| 7 | 0.62 | 9.59 | 7.52 | 8.56 | 7.8 | 17.58 |
| 8 | 0.23 | 7.52 | 5.46 | 6.49 | 5.2 | 10.70 |
| 9 | 0 | 5.46 | 2.79 | 4.12 | 2.6 | 3.94 |
| 10 | 0 | 2.79 | 0 | 1.40 | 0 | — |


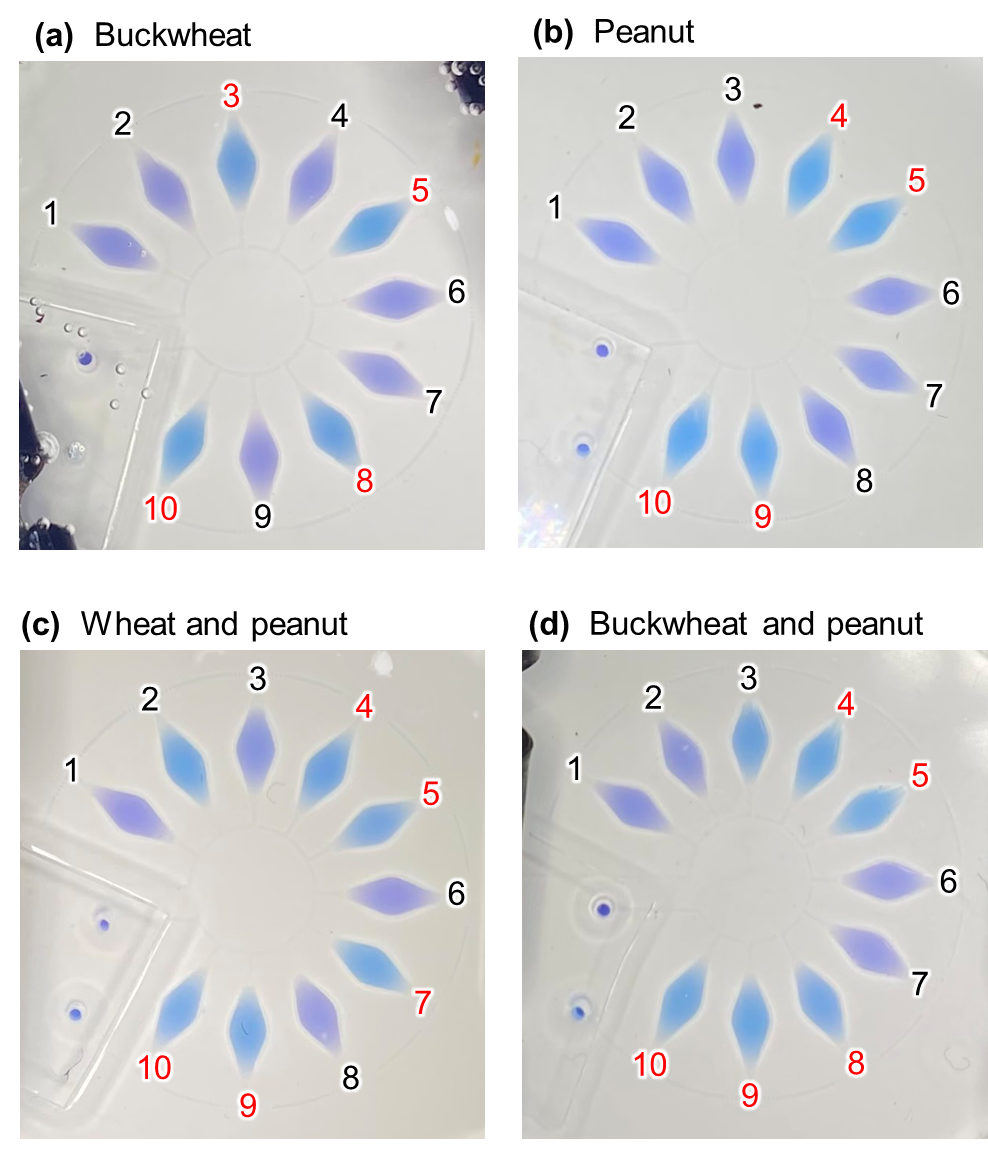


**Figure S4.** Photographs taken with a smartphone camera showing the colorimetric detection of (**a**) buckwheat DNA (Number 3 and 8), (**b**) peanut DNA (Number 4 and 9), (**c**) a mixture of wheat and peanut DNA (Number 2 and 7), and (**d**) a mixture of buckwheat and peanut DNA by LAMP assays run at 60 °C for 60 min. Universal primer sets to identify all plant species were pre-spotted in chamber Number 5 and 10 as positive controls, whereas no primers were pre-spotted in chamber Number.1 and 6 as negative controls.


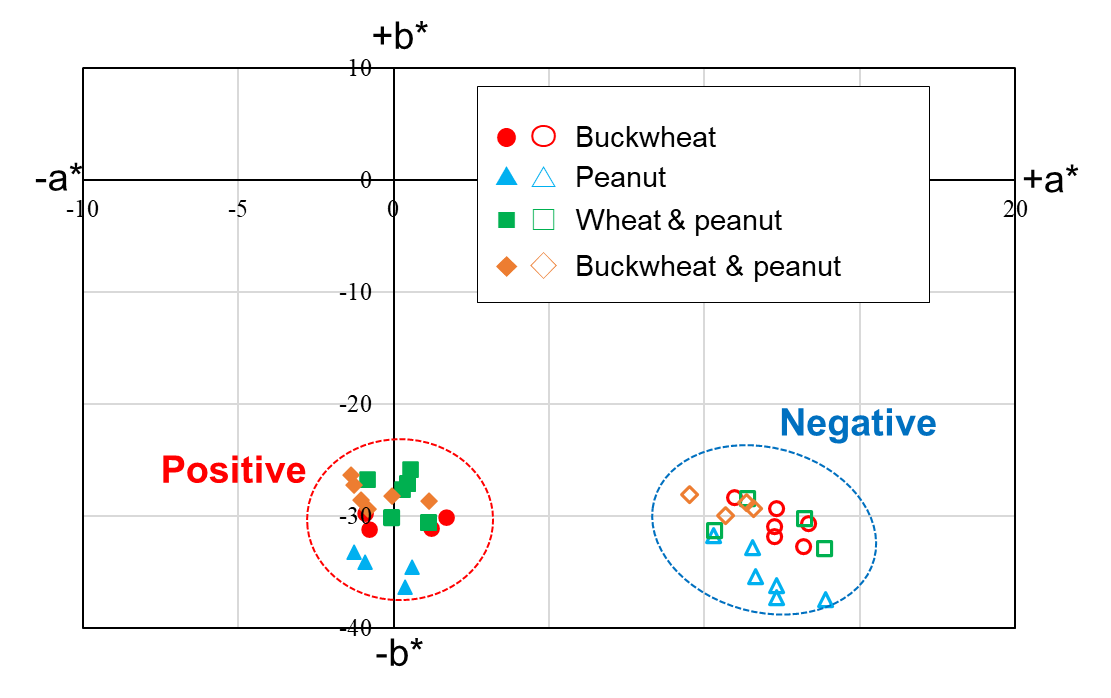


**Figure S5.** Color distributions for positive (sky blue) and negative (violet) LAMP reactions (shown in Fig. S4) run for the detection of three food allergens (wheat, buckwheat, and peanut) and tea plant as a negative control, plotted in the *a**-*b** chromatic plane of the CIELAB color space. LAMP reactions were run at 60 °C for 60 min.

**Video S1.** Sequential liquid dispensing into an array of 10 microchambers with a pair of passive stop valves including a single-faced stop valve as a temporary stop valve *S*_1_ and a double-faced stop valve as a permanent stop valve *S*_2_ at a flow rate of 20 µL/min.

**Video S2.** Sequential liquid dispensing into an array of 10 microchambers with air plug-in valves as a permanent stop valve *S*_2_, consisting of a set of two double-faced stop valves facing each other in the air exhaust microchannel, at a flow rate of 70 µL/min.

**Video S3.** Sequential liquid dispensing manually into an array of 10 microchambers with air plug-in valves at an estimated mean flow rate of ~40 µL/min.

**Video S4.** Sequential liquid dispensing into an array of 10 microchambers arranged in a circle at a flow rate of 30 µL/min.
